# Low Genetic Impact of the Roman Occupation of Britain in Rural Communities

**DOI:** 10.1093/molbev/msae168

**Published:** 2024-09-12

**Authors:** Christiana L Scheib, Ruoyun Hui, Alice K Rose, Eugenia D’Atanasio, Sarah A Inskip, Jenna Dittmar, Craig Cessford, Samuel J Griffith, Anu Solnik, Rob Wiseman, Benjamin Neil, Trish Biers, Sarah-Jane Harknett, Stefania Sasso, Simone A Biagini, Göran Runfeldt, Corinne Duhig, Christopher Evans, Mait Metspalu, Martin J Millett, Tamsin C O’Connell, John E Robb, Toomas Kivisild

**Affiliations:** Estonian Biocentre, Institute of Genomics, University of Tartu Tartu 51010, Estonia; St John's College, University of Cambridge, Cambridge CB2 1TP, UK; McDonald Institute for Archaeological Research, University of Cambridge, Cambridge CB2 3ER, UK; McDonald Institute for Archaeological Research, University of Cambridge, Cambridge CB2 3ER, UK; Alan Turing Institute, British Library, London NW1 2DB, UK; McDonald Institute for Archaeological Research, University of Cambridge, Cambridge CB2 3ER, UK; Institute of Molecular Biology and Pathology, IBPM CNR, Rome 00185, Italy; McDonald Institute for Archaeological Research, University of Cambridge, Cambridge CB2 3ER, UK; School of Archaeology and Ancient History, University of Leicester, University Road, Leicester LE1 7RH, UK; McDonald Institute for Archaeological Research, University of Cambridge, Cambridge CB2 3ER, UK; Cambridge Archaeological Unit, Department of Archaeology, University of Cambridge, Cambridge CB3 0DT, UK; Estonian Biocentre, Institute of Genomics, University of Tartu Tartu 51010, Estonia; Core Facility, Institute of Genomics, University of Tartu, Tartu 51010, Estonia; Core Facility, Institute of Genomics, University of Tartu, Tartu 51010, Estonia; Core Facility, Institute of Genomics, University of Tartu, Tartu 51010, Estonia; Department of Archaeology, University of Cambridge, Cambridge CB2 3DZ, UK; Museum of Archaeology and Anthropology, Cambridge CB2 3DZ, UK; Estonian Biocentre, Institute of Genomics, University of Tartu Tartu 51010, Estonia; Institut de Biologia Evolutiva (UPF-CSIC), Departament de Medicina i Ciències de la Vida, Universitat Pompeu Fabra, Parc de Recerca Biomèdica de Barcelona, 08003 Barcelona, Spain; Department of Human Genetics, KU Leuven, 3000 Leuven, Belgium; FamilyTreeDNA, Gene by Gene, Houston, TX 77008, USA; Wolfson College, University of Cambridge, Cambridge CB3 9BB, UK; Department of Archaeology, University of Cambridge, Cambridge CB2 3DZ, UK; Estonian Biocentre, Institute of Genomics, University of Tartu Tartu 51010, Estonia; Faculty of Classics, University of Cambridge, Cambridge CB3 9DA, UK; Department of Archaeology, University of Cambridge, Cambridge CB2 3DZ, UK; Department of Archaeology, University of Cambridge, Cambridge CB2 3DZ, UK; Estonian Biocentre, Institute of Genomics, University of Tartu Tartu 51010, Estonia; McDonald Institute for Archaeological Research, University of Cambridge, Cambridge CB2 3ER, UK; Department of Human Genetics, KU Leuven, 3000 Leuven, Belgium

**Keywords:** genomics, ancient DNA, kinship, population genomics, Roman, United Kingdom

## Abstract

The Roman period saw the empire expand across Europe and the Mediterranean, including much of what is today Great Britain. While there is written evidence of high mobility into and out of Britain for administrators, traders, and the military, the impact of imperialism on local, rural population structure, kinship, and mobility is invisible in the textual record. The extent of genetic change that occurred in Britain during the Roman military occupation remains underexplored. Here, using genome-wide data from 52 ancient individuals from eight sites in Cambridgeshire covering the period of Roman occupation, we show low levels of genetic ancestry differentiation between Romano-British sites and indications of larger populations than in the Bronze Age and Neolithic. We find no evidence of long-distance migration from elsewhere in the Empire, though we do find one case of possible temporary mobility within a family unit during the Late Romano-British period. We also show that the present-day patterns of genetic ancestry composition in Britain emerged after the Roman period.

## Introduction

At its height, the Western Roman Empire controlled a significant portion of continental Europe, including Britain. Estimates of the population of Roman Britain vary between 2.8 and nearly 4 million people over the period of 70 to 400 CE (Alcock 2011), of which rural communities accounted for about 90% (Millett 1990). Although rural local populations of this period are archaeologically well documented, their movements are less well understood and invisible in the textual record. The most visible individuals in Roman Britain are soldiers and administrators, many of whom came from other parts of the Empire (Eckardt and Müldner 2014). In general, soldiers were posted to areas away from their homelands to avoid conflicts of loyalty (Haynes 2013). Migration from the rest of the Empire into Britain was likely dominated by these groups, along with traders and the highest impact would have been in urban and military areas. Although they were extensively networked, rural communities were arguably little affected by migration (Smith et al. 2016). The extent of mobility in this period has been the subject of recent debate, with work largely focusing on the use of isotope data (Eckardt and Müldner 2014). Results from such studies indicate high levels of mobility, with 30% to 50% of individuals having non-local childhoods (Schweissing and Grupe 2003; Prowse et al. 2007; Eckardt 2010). However, as sampling for isotope analysis has been dominated by the examination of military and urban areas and from burials that may not be representative of the general population (Eckardt and Müldner 2014), our knowledge of the scale of migration and its impact on the overall population is difficult to assess.

From the genetic perspective, the subsequent Early Medieval period (5th to 10th centuries CE) resulted in a major shift toward higher affinities to Dutch, Danish, and other continental North Sea zone ancestries in eastern England, at the scale of 38% to 75% on average (Leslie et al. 2015; Schiffels et al. 2016; Gretzinger et al. 2022). It is not clear whether this is due to migration solely during the Early Medieval period, or if any change can be ascribed to gene flow during the Roman period (Oosthuizen 2017). The presence of burial goods from Britain in late Roman sites in north-west Germany (Swift 2010) indicates movement from Britain to the continent, but the same pattern is not necessarily seen in reverse. The long-standing ties between Britain and Gaul (a region encompassing modern-day Belgium, France, Luxembourg, as well as parts of Switzerland, the Netherlands, Northern Italy, and Germany [Champion 2016, pg. 155]), both prior to and during the Roman period, may obscure the genetic distinction between local, indigenous Britons, and incoming individuals.

In contrast to recent genomic studies on demographic changes during the Bronze and Iron Age (Patterson et al. 2021) and Early Medieval (Gretzinger et al. 2022) periods in Great Britain, to date, few genomes from the Roman period have been published. A study of seven individuals from a cemetery in York with decapitations showed most individuals had a higher affinity to the modern Welsh than modern English, yet also highlighted the cosmopolitan nature of the Roman empire by identifying an individual with Middle Eastern/North African ancestry (Martiniano et al. 2016). However, York was a cosmopolitan urban center and cannot be taken as typical of the province as a whole (Ottaway 2004). Another recent study showed an isolated burial at Offord Cluny, in rural Cambridgeshire, to be a male individual with Sarmatian ancestry (Silva et al. 2024). Again, the burial was not from one of the larger, formal cemeteries that may be more representative of the long-standing local population.

In general, the area that is today Cambridgeshire provides an extensively researched rural, agricultural region that is not atypical of the province as a whole (Smith et al. 2016), and thus genetic information from communities in this region provide a key opportunity to improve our understanding of the make-up of the local population(s) of Roman Britain. Here, we examine the impact of Roman occupation on rural communities in Britain by studying genome-wide data from six Roman-era sites in Cambridgeshire, with matching isotope data from three.

## Results

**Fig. 1.**
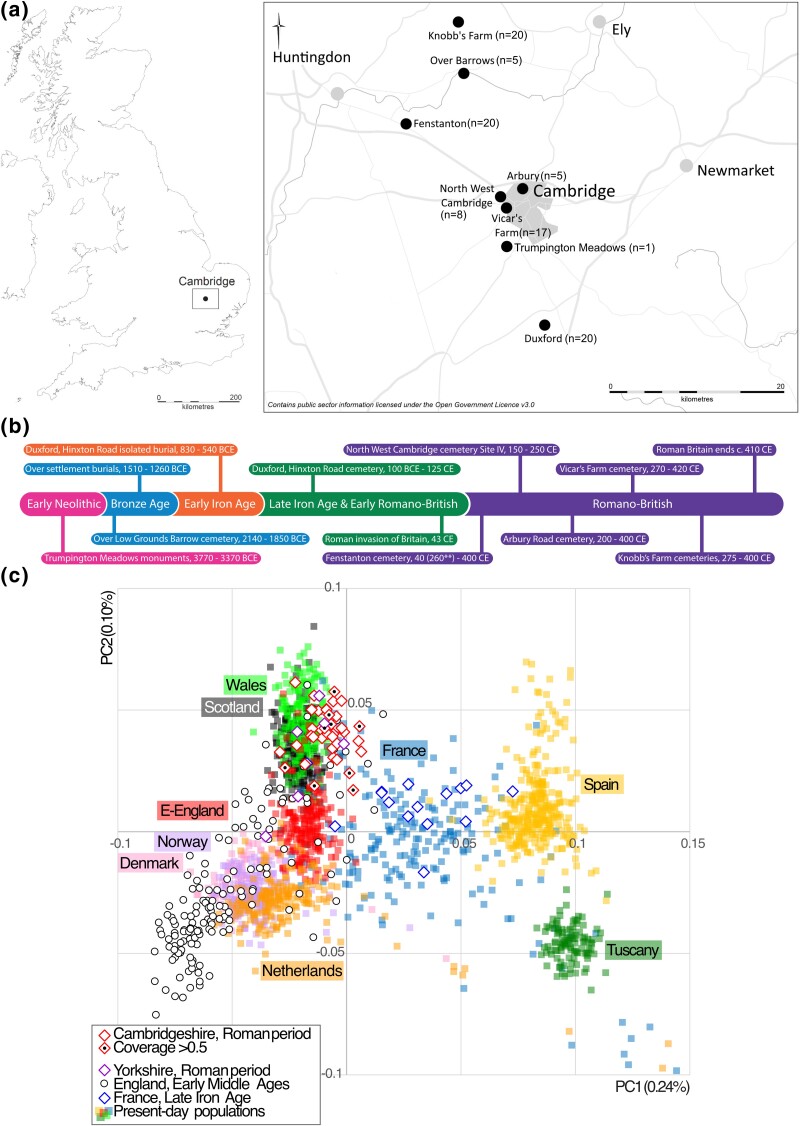
Geographical and chronological distribution of the dataset and population affinities. a) Site map modified from a map made by Vicki Herring for the After the Plague project. b) Timeline of archaeological sites and historical events covering the period of the study. c) PCA based on a selection of 1,682 present-day individuals from the UK Biobank, 1000 Genomes Project, and imputed ancient genomes, including 33 Late Iron Age/Roman genomes from Cambridgeshire (this study) and 6 from York (Martiniano et al. 2016), 15 Late Iron Age genomes from France (Fischer et al. 2022), and 150 Early Medieval genomes from England (Gretzinger et al. 2022). Two previously published Roman period genomes with genome with Near Eastern (3DRIF-26, Martiniano et al. 2016) and North Caucasus ancestry (Offord Cluny, Silva et al. 2024) were not included in the analysis.

To explore the question of the impact of migration outside of cosmopolitan centers, we generated genome-wide data for 96 ancient individuals. To provide genetic background to the region and period in this sample set, we included genome-wide data from three individuals (one newly generated) from an Early Neolithic site (3,770 to 3,370 BCE [Scheib et al. 2019], two from the nearby Bronze age site of Over Barrows (2,140 to 1,260 BCE), and one isolated Early Iron Age burial (830 to 540 BCE) from a predominantly Roman period site (Table 1). The Roman-era sites encompass six locations in the Cambridgeshire region (Fig. 1a) with occupation dates spanning 100 to 400 CE (Table 1, supplementary Data S1b, Supplementary Material online) and include farmsteads and a cemetery with a number of burials with decapitations (Wiseman et al. 2021 [see Materials and Methods]).

**Table 1:** Summary of sites and samples included in this study

Site	Time period	Inh. (N)	Crem. (N)	Genomes Available	New Genomes	Source
Trumpington Meadows monuments	3,770 to 3,370 BCE	4^a^	0	3	1	Scheib et al. 2019, This study
Over Low Grounds Barrow cemetery	2,140 to 1,850 BCE	9	44	3	1	Olalde et al. 2018, This study
Over settlement burials	1,510 to 1,260 BCE	2	0	2	1	Olalde et al. 2018, This study
Duxford, Hinxton Road isolated burial	830 to 540 BCE	1	0	1	1	This study
Duxford, Hinxton Road cemetery	100 to 125 CE	30	5	19	19	This study
North West Cambridge cemetery Site IV	150 to 250 CE	11	0	8	8	This study
Arbury Road cemetery	200 to 400 CE	6	0	5	5	This study
Vicar's Farm cemetery	270 to 420 CE	29	1	17	17	This study
Knobb's Farm cemeteries	275 to 400 CE	52	0	20	20	This study
Fenstanton, Cambridge Road/Dairy Crest cemetery	40 (260 CE)^b^ to 400 CE	48	3	20	20	This study

Inh., Inhumations; Crem., Cremations; genomes available (N) indicates total individual genomic data including previously published genomes.

^a^Skeletons 1 and 3 are almost certainly the same individual; thus, the likely total number of individuals is only three even though four were reported in the original site report.

^b^Core occupancy date is likely starting at 260 CE.

We sequenced these genomes to an average genome-wide coverage of up to 3.8× (median 0.037×). Of these, 41 were > 0.05× and used for imputation-based allele frequency analyses (supplementary Data S1a, Supplementary Material online). A subset of 33 genomes which had autosomal coverage > 0.1× were used in genome-wide autosomal genotype-related analyses. Mitochondrial haplogroups could be determined (coverage > 2×) for 66 individuals. In general, the genomes represent an equal distribution of males and females, as determined genetically, and a range of juveniles and adults of all ages (supplementary Data S1a, Supplementary Material online). The average endogenous human DNA content varies by site, with a mean of 12.03%, and genome-wide coverage 0.13×. The median estimated contamination rates from mitochondrial DNA (mtDNA) using two methods (Fu et al. 2013; Jones et al. 2015) is 0.43% and the average misincorporation of *C* > *T* in the first five base pairs (bp) is 8.11% (supplementary Data S1a, Supplementary Material online). This range of these values is typical for ancient DNA.

### Population Structure of Iron Age/Roman Cambridge

We studied the ancestry of 33 Late Iron Age/Roman period (LIA/RP) genomes from Cambridgeshire in the context of available ancient genomes from Britain and modern genomes from Europe and the Middle East using Principal Component Analysis (PCA [supplementary Data S2, Supplementary Material online]). We found that LIA/RP genomes from Cambridgeshire all draw their genetic ancestry from Western Europe (Fig. 1c) and that, like the majority of LIA/RP genomes from York, they cluster more closely with modern Welsh than local East England genomes (Fig. 1c). Unlike the two previously detected outliers, Offord Cluny from Cambridgeshire (Silva et al. 2024) and 3DRIF-26 from York (Martiniano et al. 2016), we do not detect outliers among the 33 Cambridgeshire LIA/RP genomes with >0.1× coverage examined. All Roman period populations examined show homogeneity in their North/West European ancestry in relation to external reference populations in PCA analyses based on imputed data (Fig. 1, supplementary fig. S1, Supplementary Material online) or projections made from haploid genotype calls (supplementary fig. S1, Supplementary Material online).

**Fig. 2.**
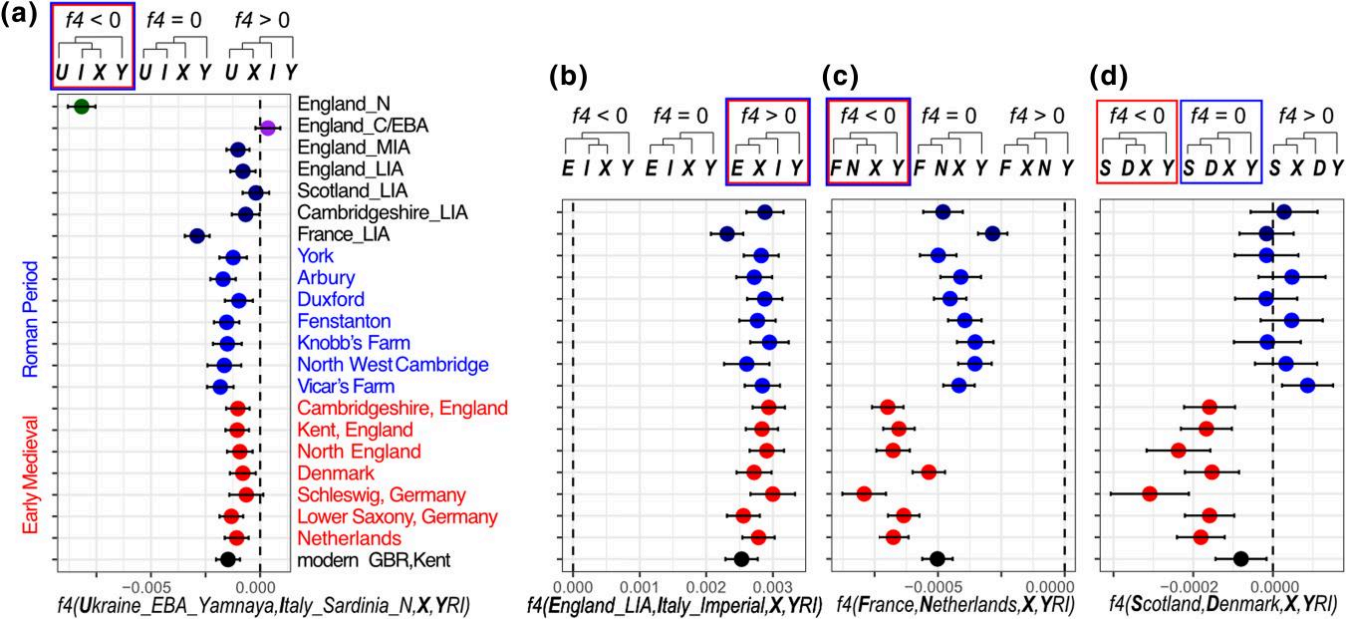
Genetic affinities of Roman period sites in England to ancient and modern populations of Europe. A-B: affinities to ancient genome groups of individuals from the Allen Ancient DNA Resource v54 (Mathieson et al. 2018; Antonio et al. 2019; Fernandes et al. 2020; Marcus et al. 2020; Patterson et al. 2021; Gretzinger et al. 2022; Mallick et al. 2024). C-D: affinities to groups of 200 individuals from the UK Biobank born in France, Netherlands, Denmark, and Scotland. Each plot shows the estimated *f4* value with an error range of two standard deviations. Respective f4 plots by individuals of the Roman sites are shown in supplementary figs. S2 and S3, Supplementary Material online.

We tested whether the imputed LIA/RP genomes have different affinities to ancient and modern European populations using *f*4 statistics. Consistent with the increased Neolithic ancestry observed in Iron Age genomes from England by Patterson et al. (2021), all six Roman period sites we tested showed consistently higher drift sharing with Sardinian Neolithic genomes than genomes from Copper and Bronze Age England (−5.3 < *Z* < −2.4; Fig. 2a). All sites show higher affinity to Late Iron Age England than to Imperial LIA/RP genomes (Fig. 2b). Unlike the Roman period York cemetery, which included burial of a long-distance migrant from the present-day Middle East or North Africa (Martiniano et al. 2016), we find no evidence of long-distance migration from the Mediterranean region among the 33 imputed genomes from Roman Cambridgeshire that we tested (supplementary fig. S2a to c, Supplementary Material online). The Cambridgeshire genomes are also not differentiated by their affinity with Late Iron Age genomes from France, Scotland, and England (supplementary fig. S2d and e, Supplementary Material online).

As previously reported in Roman period genomes from York (Martiniano et al. 2016), we find higher affinity of the Cambridgeshire LIA/RP genomes to present-day Dutch than French genomes (Fig. 2c, supplementary Data S3, Supplementary Material online). We also find that, unlike the later Early Medieval genomes, the Roman period genomes are not more similar to modern Danish than modern Scottish genomes (Fig. 2d). Nor do we observe any notable individual deviations from the patterns observed at site level (supplementary fig. S3a and b, Supplementary Material online). We observe relatively little difference in the affinities of the LIA/RP genomes to present-day groups from East and South England. A third of the Roman period individuals from Cambridgeshire (East England) show minor, but significantly higher affinity to present-day Kent than average present-day genomes from East England (supplementary fig. S3c, Supplementary Material online).

**Fig. 3.**
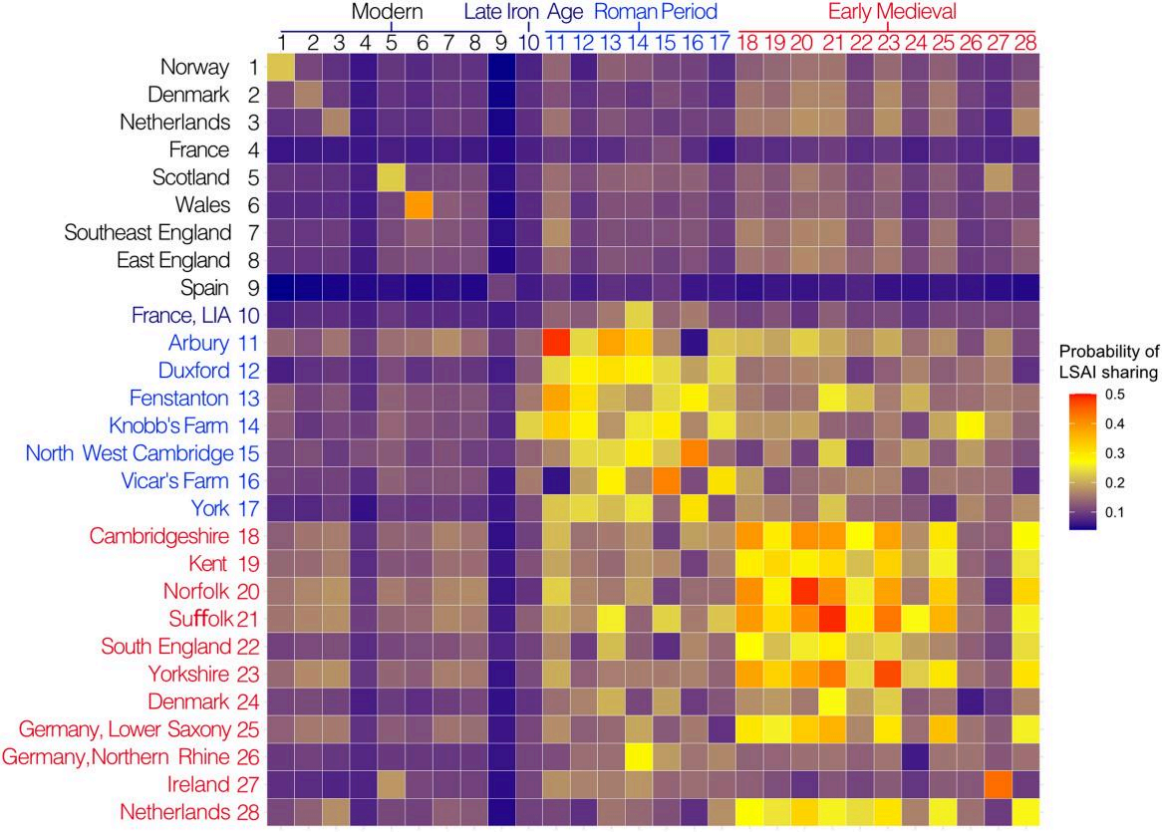
Probabilities of LSAI sharing among populations. Heatmap of probabilities of individuals from a population in a row to share at least one LSAI segment > 4 cM with individuals from populations by columns. Present-day population data from the UK Biobank, ancient imputed genomes include Late Iron Age of France (Fischer et al. 2022), Roman period data from Cambridgeshire (this study), York (Martiniano et al. 2016), and Early Medieval data (Gretzinger et al. 2022).

We further examined patterns of long shared allele intervals (LSAI) between imputed genomes of Roman individuals from Cambridgeshire, in the context of available Roman period data from York, Late Iron Age France (Fischer et al. 2022), and Early Medieval West Europe (Gretzinger et al. 2022) as well as UK Biobank data for individuals born in the UK and elsewhere in Europe (supplementary Data S4, Supplementary Material online, Fig. 3). Similar to identity-by-descent (IBD) segments, LSAIs are expected to provide a computationally tractable way to detect fine-scale structure in large cohorts (Kivisild et al. 2021). Because stretches of shared alleles in an unphased context at lengths > 4 cM are unlikely to always correspond to shared haplotypes (Freyman et al. 2020), it is meaningful to distinguish LSAIs from IBD.

Unsurprisingly, we find a relatively high level of LSAI sharing among geographically close Roman sites in Cambridgeshire, with an average probability of 25% of individuals from one site sharing an LSAI segment longer than 4cM with individuals from another site, which is more than twice as high as observed sharing among present-day individuals from East or Southeast England (Fig. 3, supplementary Data S4, Supplementary Material online). Notably, LSAI sharing among Early Medieval sites from across England (on average 32%) is higher (*P* = 0.002 by two-tailed *t*-test) than sharing among Roman sites in Cambridgeshire alone (on average of 25%), remaining high for the English Early Medieval sites across the Channel with Early Medieval sites from Lower Saxony and the Netherlands (28%). Compared to Roman sites, the Early Medieval sites from East England show (*P* = 5 × 10^−7^) increase in LSAI sharing with present-day Scandinavian and Dutch genomes from approximately 10% to 15%, which is consistent with the major increase in that period of continental northern European ancestry detected by Gretzinger et al. (2022). At the same time, LSAI sharing with Late Iron Age France drops in Cambridgeshire from the mean of 15.5% in the Roman to 10% in the Early Medieval and 8% in present-day East England which is comparable to the level of sharing between modern French and English (6.27% [supplementary Data S4, Supplementary Material online]).

To assess the extent of inbreeding in the Roman-era populations, we calculated runs of homozygosity (ROH) using HapROH (Ringbauer et al. 2021). Using a two-tailed Student's *t*-test, we find no difference in the average sum of ROH segments greater than 4cM or 8cM between the Roman-era sites (supplementary Data S5, Supplementary Material online). Nor, do we find a difference between the two newly generated Bronze Age (Over Barrows) individuals and the Roman-era populations (supplementary Data S5, Supplementary Material online).

### Genetic Kinship Structure

**Fig. 4.**
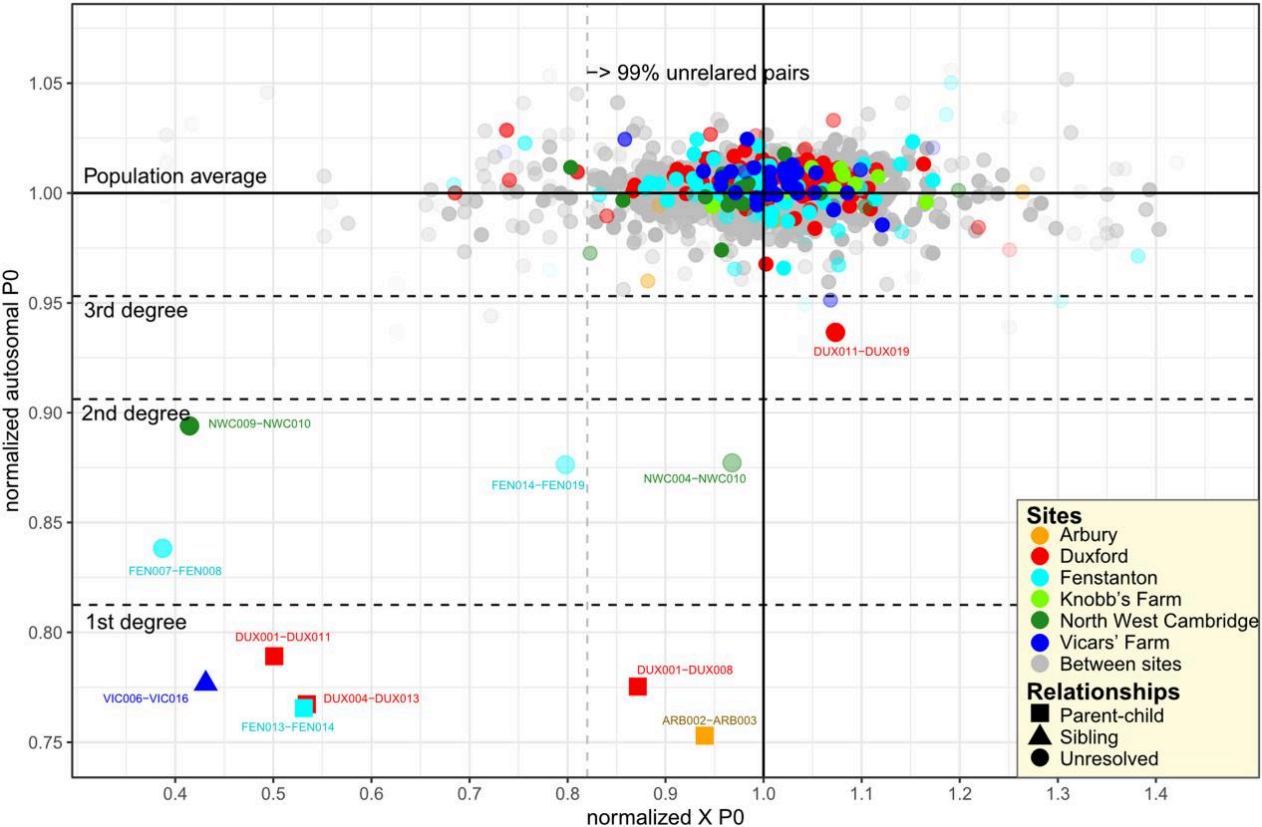
Relatedness between ancient Iron Age/Roman genomes. Degrees of relatedness, relationship types, and normalized autosomal mismatch probabilities were estimated with KIN (Popli et al. 2023). Each dot shown on the plot represents a pair of ancient genomes assessed for their mean pairwise differences divided by population average. The degree boundaries for autosomal relatedness are shown according to cutoffs defined by Kuhn et al. (2018). The lower boundary for 99% autosomally unrelated pairs is shown on the *x*-axis for guidance of X chromosome mismatch probabilities in our sample. Dots with high transparency correspond to pairs with low aggregate SNP coverage. Labels of only first- to third-degree-related pairs supported by KIN's Log-likelihood ratio > 1 and with more than 5,000 overlapping SNPs are shown.

We examined relatedness within and among the Roman sites of Cambridgeshire using Kinship INference (KIN) (Popli et al. 2023) and relationship estimation from ancient DNA (READ) (Monroy Kuhn et al. 2018) to detect first- to third-degree-related pairs of individuals (supplementary Data S1c and d, Supplementary Material online). We also used an IBD-based approach (Seidman et al. 2020) on imputed genomes to explore more distant forms of relatedness. Despite our relatively small sample sizes per site, we observed closely related pairs (Fig. 4) in all Roman age sites from Cambridgeshire except for Knobb's Farm (supplementary Data S1c, Supplementary Material online). Perhaps interestingly, both Knobb's Farm and the previously studied Driffield Terrace in York (Martiniano et al. 2016), which also did not reveal related pairs, are sites where decapitated burials are common. None of the pairwise comparisons between sites identified individuals related closer than the third degree.

Notably, within the relationships detected within the sites, we find several triangular cases of relatedness with a female individual involved in more than one pair (e.g. Duxford DUX011 [female] related with DUX019 [male] and DUX001 [male]), or in the case of North West Cambridge, we find a relationship between three sampled male individuals (NWC004, NWC010, and NWC009), who appear to be related to each other through unsampled female(s) (either not buried in this cemetery or not sampled). This is inferred by the fact that their pairwise X chromosomal differences are lower than the population average despite all carrying different mtDNA lineages (Fig. 4, supplementary Data S1f, Supplementary Material online). Genetically related individuals appear not to be clustered or buried next to each other: for example, the members of a Duxford family DUX011 (mother), DUX008 (father), and their son (DUX001) are all buried in different groups of burials (supplementary fig. S4, Supplementary Material online) identified in the original site report (Lyons 2011). Similarly, in Vicar's Farm, related pairs of individuals were buried in different groups of burials (Evans and Lucas 2020 [pages 333 to 34 & 377]).

To further explore the sharing of long LSAI (IBD) segments within and among Late Iron Age and Roman sites in Cambridgeshire, we used identical by descent via identical by state (IBIS). In all pairs of imputed individuals that were identified with READ and KIN as closely related, we found multiple LSAI segments supporting their close relatedness (supplementary Data S4, Supplementary Material online). However, in all cases, the observed total LSAI shared was less than expected from the first- to third-degree relationship, suggesting that capturing long tracts of LSAI at low coverage is hindered by fragmentation due to imputation errors. Besides the kinship pairs already detected with KIN (Fig. 4), we did not find any new relationships with IBIS within the sites. We did, however, detect a case of distant relatedness between DUX019 from Duxford and a previously reported sample 12884A (HI2, Schiffels et al. 2016) from Hinxton, who share five LSAI segments longer than 7cM consistent with an estimated kinship coefficient suggesting sixth-degree relatedness (supplementary Data S4c, Supplementary Material online). Given that the Duxford and Hinxton sites are located only 3 km from each other and are both in the Cam valley, this finding points to local mobility between geographically adjacent sites.

### Diversity of Uniparental Markers

To determine variation in the paternal lineages, we called the genotypes of 161,140 Y chromosome haplogroup informative binary markers in 30 males from the Early Neolithic, Late Iron Age, and Roman Cambridgeshire with Y chromosome coverage > 0.003× (supplementary Data S1a and e, Supplementary Material online). All individuals could be assigned to haplogroups common in modern-day Europe (supplementary Data S1f and g, Supplementary Material online). The majority (85%) belong to haplogroup R1b (supplementary Data S1f, Supplementary Material online), which became the predominant male lineage in Britain after the spread of the Beaker complex (Olalde et al. 2018). Two first-degree-related individuals from Duxford and the newly sequenced individual from Trumpington Meadows fall into the I2 clade, which captures all previously known Y chromosome lineages in Britain before the Bell Beaker Culture (supplementary Data S1e and f, Supplementary Material online). It is not clear, however, whether this particular lineage (I2-Y3722) of the Duxford father-son pair reflects local continuity and survival from a pre-Beaker population, or more recent migration, as its present-day distribution is mainly focused on Ireland with only rare cases detected in England and Scotland (https://www.yfull.com/tree/I-Y3722/). Among R1b individuals with >0.01× coverage, we identify distinct subclades, including the British/Irish Bell Beaker signature lineage R1b2–L21 (Patterson et al. 2021) as well as lineages from clades such as R1b11-Z2103 and R1b18-S1194, which have not been reported in Britain in the context of earlier time periods. Notably, none of the four R1b samples with > 0.2× Y chromosome coverage fall into the same sub-clade. Some of the identified subclades of R1b appear to be rare in a large, high-resolution modern Y chromosome compendium of more than 60,000 FamilyTreeDNA customers (supplementary Data S1f, Supplementary Material online). Overall, compared to the Copper/Bronze Age periods, we do not detect in our Roman Cambridgeshire individuals any notable changes in the composition of the Y chromosome haplogroups apart from a single I1 (NWC010) and a single G2a (DUX006) lineage that, by their presence in the Iron Age data (Patterson et al. 2021), were likely introduced to Britain from the mainland during the Iron Age (supplementary Data S1e, Supplementary Material online).

We determined mitochondrial (mtDNA) haplotypes for 66 individuals with mtDNA coverage over 2× (supplementary Data S1a and h, Supplementary Material online) and found high diversity (55 unique haplogroups). We found identical mtDNA lineages in the cases of close autosomally defined kinship (*n* = 4 [supplementary Data S1a, Supplementary Material online]) and 11 overall haplogroup matches (including close kinship). Upon close inspection of private mutations, all mtDNA haplogroup matches between individuals who were not closely related by autosomal data turned out to be different mtDNA haplotypes (supplementary Data S1a, Supplementary Material online). Overall, we find mtDNA haplogroups typical to Western Europe (supplementary Data S1f, Supplementary Material online) with little differentiation over time, particularly in comparison to major Y chromosome haplogroup shifts. The observation that Iron Age British tribes practiced polyandry, particularly within family groups (e.g. brothers), has been attributed to Julius Caesar himself (Edwards 1917). If this had been a common practice, we would expect lower diversity in mtDNA. This is not what we observe, however, given the limited size of our data, we cannot formally test this.

### Mobility Through Isotopic Analysis

As ancestry itself cannot directly confirm an individual's mobility, to further explore childhood origins and geographic mobility, we generated oxygen isotope ratio data from the tooth enamel of individuals from two sites of this study. Oxygen isotope ratio data was already published from Knobb's Farm (Wiseman et al. 2021). The oxygen isotope composition of local water sources is largely determined by the local climatic conditions (Dansgaard 1964; Pederzani and Britton 2019) and the oxygen isotope ratios measured in archaeological human tooth enamel are a reflection of the water consumed during the formation of the enamel during childhood (DeNiro and Epstein 1978; Longinelli 1984; Luz et al. 1984a, 1984b). A mismatch between enamel ratio values and estimated local values might indicate a non-local childhood (Pederzani and Britton 2019).

We measured the carbonate oxygen isotope ratios (δ^18^O_CO3_) of 32 s premolars from 17 individuals (1 Early Iron Age, 1 Middle Iron Age, and 15 Late Iron Age/Early Roman) from Duxford and 15 individuals (all Mid-Late Roman) from Vicar's Farm, and we compared the results to published data from 33 individuals from Knobb's Farm (1 Middle Iron Age, 32 Late Roman [Wiseman et al. 2021]). Due to the variation in teeth analyzed between studies, the data will not represent exactly the same period of life; however, the datasets are comparable for our purposes (Lightfoot in Wiseman et al. 2021, p. 160).

**Fig. 5.**
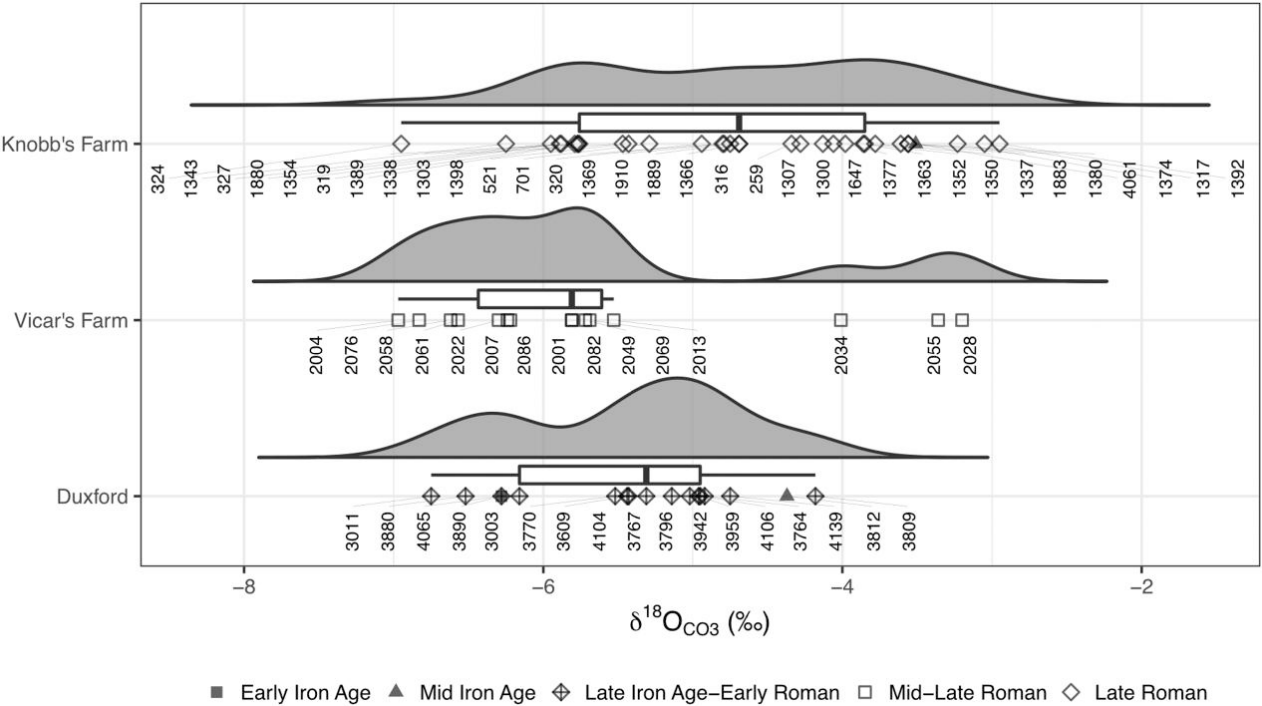
Raincloud plot of δ^18^O_CO3_ values from Duxford, Vicar’s Farm and Knobb’s Farm, showing probability distribution, median, interquartile range (IQR), outliers, and scatter of data, with individual skeleton numbers. For ID cross reference, see supplementary Data S1a, Supplementary Material online. Data for Knobb's Farm sourced from Wiseman et al. (2021).

The δ^18^O_CO3_ values across the three sites are wide-ranging and overlapping (Fig. 5;  supplementary Data S7, Supplementary Material online). Converting the δ^18^O_CO3_ values to phosphate oxygen isotope values (δ^18^O_PO4_ [Coplen 1988; Chenery et al. 2012]) allows for broad comparisons with previously published data and expected “local” environmental values. The mean δ^18^O_PO4_ value for archaeological populations from Eastern Britain has been estimated at 17.2‰ ± 1.3 (2SD [Evans et al. 2012]). The majority of isotope values for Knobb's Farm (mean: 17.2‰ ± 2.2) fall well within this estimate, while the values for Duxford (mean: 16.5‰ ± 1.6) and Vicar's Farm (mean: 16.2‰ ± 2.6) are slightly lower but with most still falling within the estimated “Eastern” range. Skeleton no. 2004 (δ^18^O_PO4_ = 14.8‰), a Mid-Late Roman male (supplementary Data S1, Supplementary Material online) from Vicar's Farm and skeletons 324 (δ^18^O_PO4_ = 14.8‰), a Late Roman male (supplementary Data S1, Supplementary Material online) and 1,392 (δ^18^O_PO4_ = 19.1‰), another Late Roman male (supplementary Data S1, Supplementary Material online), from Knobb's Farm have δ^18^O_PO4_ values that are on the edge or beyond the overall total range of values currently estimated for Britain (Evans et al. 2012; Lightfoot and O’Connell 2016). These present the most likely candidates for being longer-distance “non-locals”—skeletons 2004 and 324 may have spent their childhoods somewhere with a colder, wetter climate than Cambridgeshire and skeleton 1,392 may have spent their childhood in a warmer, drier environment.

We investigated the presence of potential outliers further, following Lightfoot and O’Connell (2016;  supplementary Data S7a, Supplementary Material online). The 1.5IQR method is considered most robust in this instance and identifies outliers only at Vicar's Farm: skeletons 2,028, 2,034, and 2,055. However, these are within the overall range of values seen at Knobb's Farm and may only appear as outliers due to small sample sizes. Statistical comparisons of all sampled individuals from the three sites indicate that the samples were unlikely to be taken from populations with the same distributions (Kruskal–Wallis δ^18^O_CO3_: *P* = 0.005, es = 0.139), with the differences lying between Vicar's Farm and Knobb's Farm (Dunn's post-hoc with Bonferroni adj: *P*-adj = 0.007). For individuals that were confidently assigned a sex estimate of female or male, when both sex and site are considered, sex does not appear to correlate with δ^18^O_CO3_ values, but the site does (two-way ANOVA δ^18^O_CO3_ (site): *P* = 0.033, es = 0.119; δ^18^O_CO3_: *P* = 0.803, es = 0.001). There also appears to be no difference in the populations by time period when the individuals were assigned to broad date categories of Iron Age (incorporating those dated Early and Mid-Iron Age), Late Iron Age–Early Roman, and Roman (incorporating those dated to Mid-Late and Late Roman), (Kruskal–Wallis δ^18^O_CO3_: *P* = 0.516, es = −0.010).

### Changes in Allele Frequency of Genetic Variants Related to Diet, Pigmentation, and Immunity

Lastly, to investigate the phenotypic impact of potential cultural or environmental changes during the Roman period, we imputed 114 SNPs known to be involved in phenotypic traits related to diet, immunity, and pigmentation in the ancient individuals presented here and studied the allele frequencies in the frame of the regional and temporal context of a total of 277 individuals (supplementary Data S6a to d, Supplementary Material online). The temporal context data were divided into four groups, from the Mesolithic to the Roman period (supplementary Data S6a, Supplementary Material online). Within British data, from the Neolithic to present-day (1,000 Genomes GBR), we found 34 SNPs with significant allele frequency differences across time groups (supplementary Data S6b, Supplementary Material online). Consistent with previous studies (Mathieson et al. 2015; Mathieson and Mathieson 2018; Olalde et al. 2018; Saag et al. 2021; Saupe et al. 2021), we observe two major periods of allele frequency change: one after the Neolithic and the other after the Bronze Age. Most of these SNPs involve Neolithic (115 individuals here analyzed) or Chalcolithic/Bronze Age (96 individuals) groups that differ from later periods. More specifically, they include two that confer lactase persistence (rs4988235, rs182549), one involved in lipid metabolism (rs2298080), two in fatty acid metabolism (rs174546, rs174570), and one in vitamin D metabolism (rs7944926).

When focusing on differences between time groups involving Iron Age/Romans (IAR, 62 individuals, supplementary Data S6a, Supplementary Material online), we find eight SNPs with significant allele frequency differences between IAR and modern GBR. In the MCM6 locus, the two lactase-persistence SNPs show a sharp allele frequency increase after the Iron Age/Roman Period, following their earlier increase after the Bronze Age. This is consistent with recent findings related to the low frequency of the lactase gene alleles in the Bronze Age and an increase in frequency in later periods (Burger et al. 2020; Segurel et al. 2020), due to gene flow and/or selection (in relation to cultural shifts) acting on dominant traits (such as lactase persistence [Mathieson and Terhorst 2022]). Between the Roman UK (44 individuals in total) and Roman Italy (11 individuals) groups, we detect no SNPs with significant allele frequency differences. Different from the study by Kerner et al. (2021), we do not observe frequency fluctuations for the TB risk factor rs34536443, which is low in frequency from the Neolithic with no significant changes over time, reaching its present-day frequency after the Iron Age/Roman period.

## Discussion

Our population-scale results indicate that, on the whole, the region of Cambridgeshire during the period of Roman occupation was composed of genetically homogenous, local populations, with limited long-distance mobility during their lifetimes and that the large-scale movement of Roman empirical forces left little impact on the genomes of these local, rural populations. This is in stark contrast to both the York individuals (*n* = 7) of the same period where one individual was a long-distance migrant, as well as the individual with Sarmatian ancestry buried just 18 miles from our sites (Silva et al. 2024). Our work highlights the potential bias of results that can come from focusing on isolated or “unusual” burials. Here, we have focused on farms and homesteads, more reflective of the local population. From documentary records it is clear that the Roman army moved large numbers of people into and through Britain, which is especially evident at sites near military locations such as Hadrian's Wall; however, the rates at which these individuals left local offspring or died and then were buried in Britain remains to be determined.

Our genetic and isotopic results indicate a lower proportion of “non-locals” than the previously estimated 30% to 50%, although oxygen isotope analysis is much less definitive than strontium isotope analysis, which many of these estimates are based on. The majority of the individuals studied could have spent their childhoods in the local area, or at least an area with similar climatic conditions to Cambridgeshire. Particularly interesting are two individuals that aDNA analysis identified as likely to be brothers (VIC006 (sk 2028) and VIC016 (sk 2076)). They have very different δ^18^O_CO3_ values (−3.2‰ versus −6.8‰), and VIC006 has the highest δ^18^O_CO3_ value at Vicar's Farm. This could indicate that the brothers were not raised in the same geographical location. However, the overall range of the Vicar's Farm δ^18^O_CO3_ values is very similar to the other two sites and it is quite possible that the apparent bimodality is a byproduct of the small sample size, and that if a larger number of samples had been analyzed from the site, the distribution would be less bimodal and the difference between the brothers could be considered part of “normal variation” at the site. Further corroborating evidence, such as strontium isotope analysis, would be required to arrive at a more definitive interpretation.

The finding of brothers and other closely related individuals at Vicar's Farm is mirrored in all the other sites, reflecting their localized, family-based community structure. The exception is Knobb's Farm, a cemetery associated with a settlement that was possibly engaged in the processing of agricultural products and in which there are a significant number of burials missing heads or with heads severed from the body indicating decapitation pre, peri, or postmortem (supplementary fig. S5, Supplementary Material online, supplementary fig. S6, Supplementary Material online). Knobb's Farm appears to have been more broadly networked than the other local farming communities sampled here, yet distinct from the cosmopolitan urban center at York, which may explain the difference in population heterogeneity. The cemetery usage period is similar in span (±140 year) to the other sites, thus making it unlikely that the lack of close genetic kinship pairs is due to burials coming from far-removed time periods. The generally poor preservation of the site reduced the number of individuals available for genetic kinship testing, thus pairs could be missing due to lack of data. Fenstanton, the site with the only known example of crucifixion in Britain (Anon 2022), has a similar genetic kinship profile to the other farmsteads and, despite having clear evidence of Roman punishment, is distinct from Knobb's Farm.

The Roman period in East Anglia was not one of great genetic change: the major sweeps of allele frequency change occurred before or after this period. Whether it was one of great cultural change, we cannot say from our data. While polyandry is described in the early period by Caesar, by the time period studied here we find no evidence for this practice in this region. We do find support for mobility, potentially even within a family, though not nearly at such high levels as previously indicated by other isotope studies.

## Materials and Methods

### Sample Information and Ethical Statement

All skeletal elements were sampled with permission from the representative bodies/host institutions. Samples were taken and processed to maximize research value and minimize destructive sampling. Teeth were sampled from skeletons using gloves. Molars were preferred due to having more roots and larger mass, but premolars were also sampled. In general, the researchers followed the recommendations of Alpaslan-Roodenberg et al. (2021).

### Archaeological Sites and Material

#### Trumpington Meadows

This site is described in detail by Scheib et al. (2019) and Evans et al. (2018). Burial 243 skeleton 2 was an isolated mandible found opposite a conglomeration of two (or possibly more) individuals (previously published by Scheib et al. 2019). The two previously published individuals from the site were brothers and while the newly sequenced individual shares the same Y chromosome lineages, he is not estimated to be closely related (below third degree) to them.

#### Over Barrows

At the Over Low Grounds site 13 km northwest of Cambridge, excavated in 2008 by the Cambridge Archaeological Unit, a small Beaker period cemetery of six inhumations underlay a collared urn-associated Early Bronze Age barrow cemetery. Two of these individuals were dated 2,199 to 1,960 and 2,126 to 1,912 cal BC, and the earliest is likely to have been buried 2,140 to 1,970 cal BC (Evans et al., 2016, pp. 336 to 7). There were also some later burials of neonates dated c. 1,900 to 1,850 cal BC. Nearby were two Middle Bronze Age inhumation burials within a settlement, dated 1,511 to 1,303 and 1,449 to 1,260 cal BC (Evans et al., 2016, p. 253; Evans et al. 2016).

#### Duxford

The site off Hinxton Road, Duxford, was excavated by CAM ARC in 2,002 (Lyons 2011). Located 11 km southeast of Cambridge, it is situated on a chalk knoll overlooking a crossing of the River Granta, a tributary of the River Cam. There was an Early Iron Age crouched inhumation dated 827 to 540 cal BC and two supposedly Middle Iron Age inhumations, one dated to 386 to 111 cal BC (Lyons, 2011, pp. 10 to 12, 15 to 16), although aDNA analysis presented here indicates that these may be Late Iron Age. During the Late Iron Age, the higher ground was defined by a series of ditches that were repeatedly redug, surrounding a short-lived timber-framed rectangular shrine and a burial ground that was in operation c. 100 CE—125 CE (Lyons, 2011, pp. 38 to 49). The burials are believed to have “formed a selected part of a community perhaps largely made up of a single family or other social grouping” (Lyons, 2011, p. 38). A range of orientations and grave goods were present, with the 27 or more burials containing 37 to 8 individuals divided into four or five groups based on spatial patterning, orientation, etc. (Group 1a: six inhumations and three cremations; Group 1b: two cremations; Group 2: nine inhumations; Group 3: three inhumations; Group 4: six inhumations).

#### Vicar's Farm

Vicar's Farm is a rural settlement located 1.3 km west of the extensive Romano-British roadside settlement of Cambridge, falling in its immediate hinterland. Excavated by the Cambridge Archaeological Unit in 1999 to 2000 (Evans and Lucas 2020), there is evidence of Iron Age activity with a Romano-British settlement that commences c. 80 AD with a cremation cemetery, a small timber shrine and a farmstead with a rectilinear ditch system, aisled building, and various other enclosures. Over time the settlement expanded and c. 270 AD, an inhumation cemetery was established on the southern edge of the settlement within the ditched enclosure system (Evans and Lucas, 2020, pp. 314 to 37; Fig. 3.46). This cemetery presumably served part or all of the nearby rural settlement, which shows some signs of being of higher status than most other local settlements and may have fulfilled some minor central place role within the local rural settlement hierarchy. There is evidence that neonates were buried within the settlement itself rather than the cemetery and it is possible that high-status individuals were buried elsewhere.

The studied skeletons come from the inhumation cemetery, where thirty individuals were recovered from 29 graves. Eight individuals appear to have been buried in coffins, while hobnails indicate that seven were either wearing or accompanied by footwear. Grave goods accompanying seven individuals included bracelets, finger-rings, a glass bead necklace, ceramic vessels, and a cache of glass fragments. Most graves are orientated roughly north–south or east–west, and the burials were mainly extended and supine, with just one crouched burial.

#### North West Cambridge

Archaeological investigations at North West Cambridge by the Cambridge Archaeological Unit between 2009 and 2019 revealed a series of rural Romano-British settlements. The sampled skeletons come from settlement RB2.C (Site IV [Cessford and Evans 2014]  supplementary fig. S7, Supplementary Material online). Initially crossed by a double-ditched boundary the area was initially largely empty until it was divided into a series of ditched enclosures. An inhumation cemetery was established within one of the enclosures. This consisted of eleven definite and one possible burials, plus another burial a short distance away. These were largely of adults with some possible sub-adults and span the period c. 150 to 250 CE, although burials may have continued slightly after that time. Eleven of the burials had some evidence for coffins, ten or eleven of the burials had hobnailed shoes, and five or six were accompanied by beakers. There may have been some other grave goods although these are less certain, and there was a single decapitation burial.

#### Knobb's Farm

Excavations at Knobb's Farm, Somersham, Cambridgeshire, by the Cambridge Archaeological Unit between 2000 and 2010 uncovered three small late Roman cemeteries, positioned at the edge of a farming settlement by boundary ditches in a former field system dating to the fourth-century CE (Wiseman et al. 2021). The 52 burials found (11 individuals from eight graves in Cemetery 1; 28 individuals from 30 graves in Cemetery 2; 13 individuals from 12 graves in Cemetery 3) included 17 decapitated bodies and 13 prone burials. At least three bodies were buried in coffins, 15 were accompanied by pottery vessels with other grave goods including an antler comb, 30 beads, and the remains of a box. It has been suggested that the decapitated burials relate to judicial execution.

#### Fenstanton Cambridge Road and Fenstanton Dairy Crest

Albion Archaeology evaluated and then dug two adjacent sites at the southern edge of the village of Fenstanton, 15 km north-west of Cambridge and close to the Via Devana. The River Great Ouse runs 1.5 km to the north of the village, and the underlying geology is sand and gravels overlying mudstone. The Cambridge Road site is primarily on level pasture and lies at a height of about 7 m OD; Dairy Crest is on a former dairy site with modern buildings and hardstanding, about 4 to 15 m OD.

The open-area excavations of 2017 to 2018 (c. 5.5 ha excavated) revealed the area had late Iron Age material succeeded by a large enclosed settlement, occupied from the beginning of the Roman period and continuing into the latter half of the fourth-century; there were traces of a Late Roman timber building. It was probably primarily agricultural and contained a specialist cattle butchery and evidence of domestic, craft, and small-scale industrial activity; some above-average status occupation is suggested by fine ware, high-status artifacts, and building ceramics.

Several clusters of inhumations were found, in total containing 48 individuals, plus three cremations including a bustum. Graves were primarily NW-SE, inhumations extended or semi-flexed supine but some non-normative (prone, contracted, splayed knees, and head to SE). Many nails were within graves, suggesting coffins or biers, together with dress accessories and hobnails. One burial has apparent evidence of crucifixion.

#### Arbury

The details of this site are found in the study by Fell (1956). The remains are housed at the Duckworth Laboratory, Department of Archaeology, University of Cambridge. Five teeth were sampled for ancient DNA analysis. All individuals were middle-aged adults. Burials 1 and 4 were inside lead-lined stone coffins. Burials 1, 2, and 3 had no associated grave goods. Burial 5, which is genetically related to Burial 1, was buried in a wooden coffin and had parts of a small glass jug and a colored bowl at the base.

### Generation and Analysis of Isotopic Data

For this study, teeth from 17 individuals (1 Early Iron Age, 1 Middle Iron Age, and 15 Late Iron Age/Early Roman) from Hinxton Road, Duxford, and 15 individuals (all Mid-Late Roman) from Vicar's Farm, Cambridge, were sampled for carbonate δ^18^O analysis (δ^18^O_CO3_). Only permanent second premolars (PM2) or second molars (M2) were selected for analysis, with the enamel development of these teeth occurring between c.2.5 and 7.5 years (AlQahtani 2008).

Pretreatment of the enamel samples was carried out following a protocol based on methods in the study by Balasse et al. (2002). To remove surface contaminants, the outer surface of the tooth enamel was abraded using a handheld Dremel drill with a round-headed, diamond-tipped drill bit. Following this, approximately 5.5 to 10.0 mg of enamel powder was collected using a smaller round-headed diamond-tipped drill bit. Samples were then vortex mixed in approximately 0.1 ml per mg of a sample of 2% to 3% aq. sodium hypochlorite (NaOCl) and refrigerated for 24 h. Samples were rinsed five times with distilled water and then vortex mixed in 0.1 ml per mg of a sample of 0.1 M aq. acetic acid (CH_3_COOH) and left at room temperature for 4 h. Samples were then rinsed five times with distilled water, frozen, and placed in a freeze dryer until full lyophilization. Approximately 2 to 4 mg of the resultant enamel powder was weighed into glass gas bench tubes. For each batch of samples submitted for analysis, 2 to 4 mg of two in-house faunal enamel standards were also weighed into glass gas bench tubes (eight standard tubes in total). The glass vials were vacuum sealed, and the samples were reacted with 100% orthophosphoric acid at 90°C using a Micromass Multicarb Sample Preparation System. The CO_2_ produced was then dried and transferred cryogenically into a Gas Bench II coupled to a Delta V mass spectrometer in the Godwin Laboratory, Department of Earth Sciences, Cambridge. All results for both carbon and oxygen are measured and reported on the international scale relative to VPDB calibrated through the NBS19 standard (Coplen 1988; Hoefs). Based on repeated measurements of the international and in-house standards, the analytical error was <± 0.10‰ for δ^18^O_CO3_.

All δ^18^O values are primarily reported as δ^18^O_CO3_ (VPDB) values. Phosphate δ^18^O (δ^18^O_PO4_ [VPDB]) values have been estimated by converting δ^18^O_CO3_ (VPDB) to δ^18^O_CO3_ (VSMOW) using equation: δ^18^O_CO3_ (VSMOW) = 1.03091 × δ^18^O_CO3_ (VPDB) + 30.91 (Coplen 1988); then, converting δ^18^O_CO3_ (VSMOW) to δ^18^O_PO4_ (VSMOW) using equation: δ^18^O_PO4_ (VSMOW) = 1.0322 × δ^18^O_CO3_ (VSMOW)—9.6849 (Chenery et al. 2012). δ^18^O_PO4_ (VSMOW) values are more comparable with other datasets but each conversion does incur error (Chenery et al. 2012).

All statistical analysis and graphical representations of the results were performed using R version 4.0.3 and R Studio version 1.4.1106. Statistical analysis was primarily undertaken using R package “rstatix,” following Kassambara (2019). Where *P*-value-based null hypothesis testing was used, appropriate testing of assumptions was carried out to make sure there were no major violations of the methods and non-parametric testing was applied where appropriate. Any *P*-values generated were considered in context and making conclusions drawn primarily from *P*-values alone was avoided. Outliers were identified using three methods: >1.5 × IQR, >3 median absolute deviations (MAD) from median and >2 standard deviation (SD) from mean (Lightfoot and O’Connell 2016). Raincloud plots were produced following Allen et al. (2019), using R code by Allen et al., and the R package “cowplot.” Raincloud plots combine a “split-half violin” plot (showing the probability density), a boxplot (showing the median and interquartile range [IQR]), and a jittered raw data scatterplot.

### Sampling, Ancient DNA Extraction and Library Preparation

Tooth and petrous bone samples were processed in the clean room of the dedicated ancient DNA laboratory of the Institute of Genomics, University of Tartu, Estonia following established protocols already detailed in publicly published protocols on protocols.io. (Sampling: Keller and Scheib (2023a)), petrous portions were sampled with drill wheels and treated as teeth (Decontamination: Keller and Scheib (2023b); Extraction and purification: Keller and Scheib (2023c)). Double-stranded libraries were produced following Keller et al. (2023d) except that only single indexes were used.

### DNA Sequencing

DNA was sequenced using the Illumina NextSeq500/550 High-Output single-end 75-cycle kit. As a norm, 15 to 20 samples were sequenced together on one flow cell; additional data was generated for 34 samples to increase coverage (supplementary Data S1a, Supplementary Material online).

### Mapping

Before mapping, the sequences of the adapters, indexes, and poly-G tales occurring due to the specifics of the NextSeq 500 technology were cut from the ends of DNA sequences using cutadapt-1.11 (Martin 2011). Sequences shorter than 30 bp and quality <30 were also removed with the same program to avoid random mapping of sequences from other species.

The sequences were aligned to the reference sequence GRCh37 (hg19) using Burrows-Wheeler Aligner (BWA 0.7.12; Li and Durbin 2010) and the command *aln* with re-seeding disabled.

After alignment, the sequences were converted to binary alignment map file format and only sequences that mapped to the human genome were kept with samtools 1.3 (Li et al. 2009). Afterward, the data from different flow cell lanes were merged and duplicates were removed using picard 2.12 (http://broadinstitute.github.io/picard/index.html).

### aDNA Authentication

As a result of degradation over time, aDNA can be distinguished from modern DNA by certain characteristics: short fragments and a high frequency of C=> T substitutions at the 5′ ends of sequences due to cytosine deamination. The program mapDamage2.0 (Jónsson et al. 2013) was used to estimate the frequency of 5′ C=> T transitions. Rates of contamination were estimated on mitochondrial DNA by calculating the percentage of non-consensus bases at haplogroup-defining positions as detailed in (Jones et al. 2015). Each sample was mapped against the RSRS downloaded from phylotree.org and checked against haplogroup-defining sites for the sample-specific haplogroup.

Samtools 1.3 (Li et al. 2009) option *stats* was used to determine the number of final reads, average read length, average coverage, etc. The average endogenous DNA content (proportion of reads mapping to the human genome) was 12.03% (0.003 to 54.65%).

The depth of coverage was calculated using mosdepth (Pedersen and Quinlan 2018).

### Genetic Sex Estimation

Genetic sex was estimated using the methods and script described by Skoglund et al. (2013), from the fraction of reads mapping to Y chromosome out of all reads mapping to either × or Y chromosome. Genetic sex was estimated for libraries with a coverage > 0.01× and only reads with a mapping quality > 30 were counted for the autosomal, X, and Y chromosome.

### Determining mtDNA Haplogroups

Raw data were aligned to the Revised Cambridge Reference Sequence (Andrews et al. 1999) using the same settings as for autosomal alignment and variants called using bcftools *pileup* (Danecek et al. 2011). Mitochondrial DNA haplogroups were determined using Haplogrep2 on the command line (Kloss-Brandstätter et al. 2011). Subsequently, the identical results between the individuals were checked visually by aligning mapped reads to the reference sequence using samtools-1.3 (Li et al. 2009) command *tview* and confirming the haplogroup assignment in PhyloTree (accessed at: www.phylotree.org). Additionally, private mutations were noted for further kinship analysis.

### Y Chromosome Variant Calling and Haplotyping

A total of 161,140 binary Y chromosome SNPs that have been detected as polymorphic in previous high-coverage whole Y chromosome sequencing studies (Hallast et al. 2015; Karmin et al. 2015; Poznik et al. 2016) were called in 29 males with more than 0.003× Y chromosome coverage using ANGSD-0.916 (Korneliussen et al. 2014) “-doHaploCall” option. A subset of 144,550 sites yielded a call in at least one of the samples and in the case of 5,653 sites at least one of the 29 samples carried a derived allele (supplementary table S1E, Supplementary Material online). Basal haplogroup affiliations (supplementary table S1H, Supplementary Material online) of each sample were determined by assessing the proportion of derived allele calls (pD) in a set of primary (A, B, C…T) haplogroup-defining internal branches, as defined by Karmin et al. (2015), using 1,677 informative sites. In the case of 25/29 samples (with the exception of the four lowest coverage samples whose haplogroup affiliation could only be supported by two sites), the primary haplogroup could be determined unambiguously with the support of at least three variants in the derived state. Further detailed sub-haplogroup assignments within the phylogeny of the primary haplogroup were determined on the basis of mapping the derived allele calls to the internal branches of the FamilyTreeDNA tree based on approximately 52,500 modern high-coverage genomes (sequenced with the Big Y technology) and highlighting the marker tagging the branch with the lowest derived allele frequency (supplementary table S1F, Supplementary Material online).

### Comparative Genetic Data Used in the Analyses

Supplementary Data S2 contains details of the sources of comparative genomes used in the analyses of this work. PCA (Fig. 1c) used a selection of 1,682 present-day individuals from the UK Biobank, 1000 Genomes Project as modern references along with imputed ancient genomes: 6 Late Iron Age/Roman genomes from York (Martiniano et al. 2016), 15 Late Iron Age genomes from France (Fischer et al. 2022), and 150 Early Medieval genomes from England (Gretzinger et al. 2022). Two previously published Roman period genomes with genome with Near Eastern (3DRIF-26 [Martiniano et al. 2016]) and North Caucasus ancestry (Offord Cluny [Silva et al. 2024]) were not included in the analysis.

f4 Tests presented in Fig. 2 used three individuals from Ukraine_EBA_Yamnaya (Mathieson et al. 2018), 13 from Italy_Sardinia_N (Marcus et al. 2020), 19 England_LIA (Patterson et al. 2021), 35 Italy_Imperial.SG (Antonio et al. 2019), and modern references from the UK Biobank (Sudlow et al. 2015) with 200 each from France, Netherlands, Scotland, and Denmark.

Furthermore, the f4 tests presented in supplementary Data S3, Supplementary Material online included comparative data from: 28 England_C_EBA (Olalde et al. 2018), 19 England_LIA (Patterson et al. 2021), 20 England_N (Brace et al. 2019), 10 France_GrandEst_IA2 (Patterson et al. 2021), 35 Italy_Imperial.SG (Antonio et al. 2019), 13 Italy_Sardinia_N (Marcus et al. 2020), 11 Scotland_LIA (Patterson et al. 2021), and 3 Ukraine_EBA_Yamnaya (Mathieson et al. 2018).

LSAI analyses presented in Fig. 3 used present-day population data from the UK Biobank: 91 from Norway, 177 from Denmark, and 200 each from Netherlands, France, Scotland, Wales, Southeast and East England. Ancient imputed genomes included 15 from the Late Iron Age of France (Fischer et al. 2022), 6 from York (Martiniano et al. 2016), and 253 from Early Medieval Europe (Gretzinger et al. 2022).

### Pseudo-haploid Data

Autosomal variants were called with the ANGSD-0.921 software (Korneliussen et al. 2014) command –doHaploCall keeping base for the 597,573 positions that are present in the “1240 K + HO” dataset downloaded from David Reich Lab (https://reich.hms.harvard.edu/allen-ancient-dna-resource-aadr-downloadable-genotypes-present-day-and-ancient-dna-data, release: March 1, 2020 [Martiniano et al. 2016; Mathieson et al. 2018; Antonio et al. 2019; Fernandes et al. 2020; Marcus et al. 2020; Patterson et al. 2021; Gretzinger et al. 2022]). Files were converted to EIGENSTRAT format using the program convertf from the EIGENSOFT 7.2.0 package (Patterson et al. 2006).

### Principal Component Analysis

Two PCAs were made for this work: (i) to compare pseudo-haploid data and imputed data (supplementary fig. S1, Supplementary Material online) and (ii) to assess the ancestry of the imputed (Fig. 1c). PCA for supplementary fig. S1, Supplementary Material online was performed using the program smartpca (Patterson et al. 2006; Price et al. 2006) from EIGENSTRAT, projecting ancient genomes with coverages above 0.05× (using both imputed and pseudo-haploid genotypes) as well as the human reference genome onto PC space established using modern genomes. A genotype probability (GP) filter (MAX(GP)≥0.99) was applied to the imputed genotypes prior to projection. For Fig. 1, we used FlashPCA2 (Abraham et al. 2017) on imputed genomes (without projection) together with modern reference genomes after excluding variants in linkage disequilibrium with the PLINK –indep-pairwise 1000 50 0.5 option and exclusion of the likely non-neutral regions exclusion_regions_hg19.txt.

### Global Whole-genome Imputation

Following Hui et al. (2020), genotype likelihoods were first called using ANGSD (Korneliussen et al. 2014) with the following options:

-doMajorMinor 3 -GL 1 -doPost 1 -doVcf 1 -doMaf 1 -checkBamHeaders 0.

Then, they were updated with BEAGLE 4.1 (Browning and Browning 2016) in the -gl mode, followed by imputation in Beagle -gt mode with BEAGLE 5 (Browning et al. 2018) from sites where the GP of the most likely genotype reaches 0.99. To balance between imputation time and accuracy, we used 503 European genomes from the 1000 Genomes Project Phase 3 (1000 Genomes Project Consortium et al. 2015) as the reference panel in Beagle -gl step, and 27,165 genomes (except for chromosome 1, where the sample size is reduced to 22,691 due to a processing issue in the release) from the Haplotype Reference Consortium (HRC; McCarthy et al. 2016) in the Beagle -gt step. Because Beagle treats “./.” in the VCF input as sporadically missing and imputes them during haplotype phasing, which damages the accuracy when such missing genotypes are common, we imputed each genome individually so that missing genotypes were not included in the VCF input to Beagle 5. For downstream PCA, f4 tests, and IBIS analyses, we used imputed genotypes of 33 individuals that had > 0.1× coverage as a threshold that has been previously shown (Kivisild et al. 2021) to provide high accuracy of results.

### f4 Statistics

We computed *f4* statistics with AdmixTools v7.0.1 software qpDstat (with active F4 option) module using imputed Roman period genomes from this study along with 800 present-day genomes from the UK Biobank (Sudlow et al. 2015) and 1000 Genomes Project (1000 Genomes Project Consortium et al. 2015) and 290 ancient genomes from Europe (Martiniano et al. 2016; Mathieson et al. 2018; Antonio et al. 2019; Fernandes et al. 2020; Marcus et al. 2020; Patterson et al. 2021; Gretzinger et al. 2022).

### Kinship Analyses

A total of 5.5 million autosomal and 158 thousand X chromosome SNPs with minimum allele frequency (MAF) > 0.05 in UK10K males were used in kinship analysis. The analyses were restricted to 52 individuals with over 0.01× coverage. For the analyses with READ (Monroy Kuhn et al. 2018), variants were called with ANGSD (Korneliussen et al. 2014) command –doHaploCall. The ANGSD output files were converted to .tped format, which was used as an input for kinship analyses with READ (Monroy Kuhn et al. 2018). In addition to first- and second-degree relationships, we also estimated P0 cutoffs (15/16 = 0.9375 as per Monroy Kuhn et al. 2018) for the detection of third-degree relatives.

In KIN (Popli et al. 2023) analysis, we used the KINgaroo and KIN scripts with the default settings and reported only relationships with log-likelihood ratios > 1. In IBD analyses based on imputed genomes, we used IBIS (Seidman et al. 2020) with a 7-cM threshold to screen for cases of distant relatedness within and among sites.

### Runs of Homozygosity

We used hapROH (Ringbauer et al. 2021) to detect runs of homozygosity (ROH) in ancient genomes. A GP filter (MAX(GP)> = 0.99) was applied to the imputed genotypes prior to running hapROH. Using information from a reference panel, hapROH has been shown to work for genomes with more than 400 K of the 1,240 K SNPs panel covered at an error rate lower than 3% in pseudo-haploid genotypes (Ringbauer et al. 2021). We note that the requirement is broadly in line with the imputation accuracy we get from coverages as low as 0.05×, where ∼60% of common variants (MAF ≥ 0.05) in the HRC panel are recovered with an accuracy greater than 0.95 in diploid genotypes (Hui et al. 2020). Among common variants in the HRC panel, 853,159 overlap with the 1,240 K SNPs panel.

To construct the reference haplotypes, 1000 Genomes Project data were used. We kept the standard parameters in hapROH, which had been optimized for 1,240-K aDNA genotype data:

e_model = “haploid”, post_model = “Standard”, random_allele = True, roh_in = 1, roh_out = 20, roh_jump = 300, e_rate = 0.01, e_rate_ref = 0.0, cutoff_post = 0.999, and max_gap = 0, roh_min_l = 0.01

### LSAI Sharing and Individual Connectedness Inference

Long shared allele intervals (LSAI) and kinship coefficients were estimated from merged plink files of 61 imputed ancient genomes, 503 Europeans from the 1000 Genome Project, and UK Biobank data with IBIS version 1.20.9 using different minimum shared segment length (-min_L) threshold—4 cM for population genetic inference and 5 and 7 cM for kinship analyses—together with -maxDist 0.1 and -mt 300 parameters. In total, 269,319 binary SNPs with MAF > 0.05 were used. Probabilities of LSAI sharing among groups were estimated as by Kivisild et al. (2021).

### Phenotype Prediction

Local imputations were carried out on a dataset of 277 ancient individuals with coverage > 0.05×, a threshold which has been shown to yield heterozygote sensitivities ∼ 90% with the two-stage imputation including final filtering that keeps variants with GP > 0.99 (Hui et al. 2020). The dataset includes 43 individuals reported here for the first time, 223 previously published ancient genomes from the British Islands and 11 ancient Italian genomes analyzed for phenotypes in the study by Saupe et al. (2021 [supplementary Data S6a, Supplementary Material online]). The ancient samples span from about 8,500 BC to 400 CE. To perform the pigmentation prediction in terms of eye, hair, and skin color, we used the forensic HIrisPlex-S system (Chaitanya et al. 2018), after excluding two variants (namely, the indel rs312262906 and rs201326893 with MAF = 0 in HRC) from our analysis. For each of the remaining 39 target variants, we imputed genotypes from >2 Mb regions including the target and extracted its genotype for further analyses if its GP score was higher than 0.99. We called the variants using ATLAS v0.9.0 (Link et al. 2017) task = call and method = MLE commands at positions with a minimum allele frequency (MAF) ≥ 0.1% in the reference panel, which has been selected according to the different components of the samples: (i) Europeans from 1000 Genomes (EUR [1000 Genomes Project Consortium et al. 2015]) for Mesolithic, Neolithic, Copper Age, and Bronze Age ancient genomes (Olalde et al. 2018; Brace et al. 2019; Sánchez-Quinto et al. 2019; Scheib et al. 2019; Cassidy et al. 2020 [supplementary Data S6a, Supplementary Material online]); (ii) UK10K individuals extracted from the Haplotype Reference Consortium (HRC [McCarthy et al. 2016]; accessed at http://www.haplotype-reference-consortium.org/) for Iron Age, Roman, and Early Medieval individuals from Great Britain from present and previous studies (Martiniano et al. 2016; Schiffels et al. 2016 [supplementary Data S6a, Supplementary Material online]); (iii) EUR plus the MANOLIS (EUR-MNL) set from Greece and Crete extracted from the HRC (McCarthy et al. 2016) for the Imperial and Later Romans from (Antonio et al. 2019) and already analyzed for the same phenotypic variants in (Saupe et al. 2021 [supplementary Data S6a, Supplementary Material online). After calling the variants separately for each sample, we merged them in one multi-sample VCF file per region. We used the merged VCFs as input for the first step of our imputation pipeline (Hui et al. 2020 [genotype likelihood update]), performed with Beagle 4.1 -gl command (Browning and Browning 2016) using the same panels as before as reference (supplementary Data S6a, Supplementary Material online). We then discarded the variants with a genotype probability (GP) less than 0.99 and imputed the missing genotype with the -gt command of Beagle 5.0 (Browning et al. 2018) using the HRC as a reference panel for all groups of samples. We then discarded the variants with a GP < 0.99 and used the remaining SNPs to perform the phenotype prediction. Sample-by-sample phenotype prediction and genotype at the selected phenotype informative SNPs, reported as the number of effective alleles (0, 1, or 2) are shown in supplementary Data S6d, Supplementary Material online.

We then grouped the individuals into different cohorts depending on both time and space. First, we grouped the ancient individuals from the British Islands in five groups from the Mesolithic to the Early Medieval period (supplementary Data S6a and b, Supplementary Material online) and compared their allele frequency at phenotypic markers with those observed in modern GBR (supplementary Data S6b and c, Supplementary Material online). Allele frequencies have been estimated as (2HOM+HET)/2N, where HOM is the number of homozygous individuals for the effective allele, HET is the number of heterozygous individuals and N is the total number of individuals in each cohort (Relethford 2012). We compared the raw number of effective alleles in groups with a sample size higher or equal to 5 by performing an ANOVA test, and for the significant variants, we performed a Tukey test to identify the significantly different pairs of groups (supplementary Data S6b, Supplementary Material online). Using the same approach, we also analyzed the difference between Iron Age and Roman Britain by creating eight local groups and comparing them with ancient Roman Italians, discarding the groups with a sample size of less than 5 (supplementary Data S6a and c, Supplementary Material online). For both comparisons, we used Bonferroni's correction on an alpha value of 0.01 for the number of tested SNPs to set the significance threshold.

## Supplementary Material

msae168_Supplementary_Data

## Data Availability

The accession number for the DNA sequences reported in this paper is European Nucleotide Archive (ENA): under accession number PRJEB52707 (http://www.ebi.ac.uk/ena/data/view/PRJEB52707). The data are also available through the data depository of the Estonian Biocentre (EBC) (http://www.ebc.ee/free_data). The modern comparative datasets we used were UK Biobank (https://www.ukbiobank.ac.uk/), the Haplotype Reference Consortium (https://www.sanger.ac.uk/collaboration/haplotype-reference-consortium/), and the 1000 Genomes Project (https://www.internationalgenome.org/).
