# Supplementary material for: Low Genetic Impact of the Roman Occupation of Britain in Rural Communities
Source: Mol Biol Evol. 2024 Sep 12;41(9):msae168. doi: 10.1093/molbev/msae168 (PMC11393495; doi:10.1093/molbev/msae168)
Supplement: msae168_Supplementary_Data [file msae168_supplementary_data.zip › Supplementary Figures.pdf]

Supplementary Figures

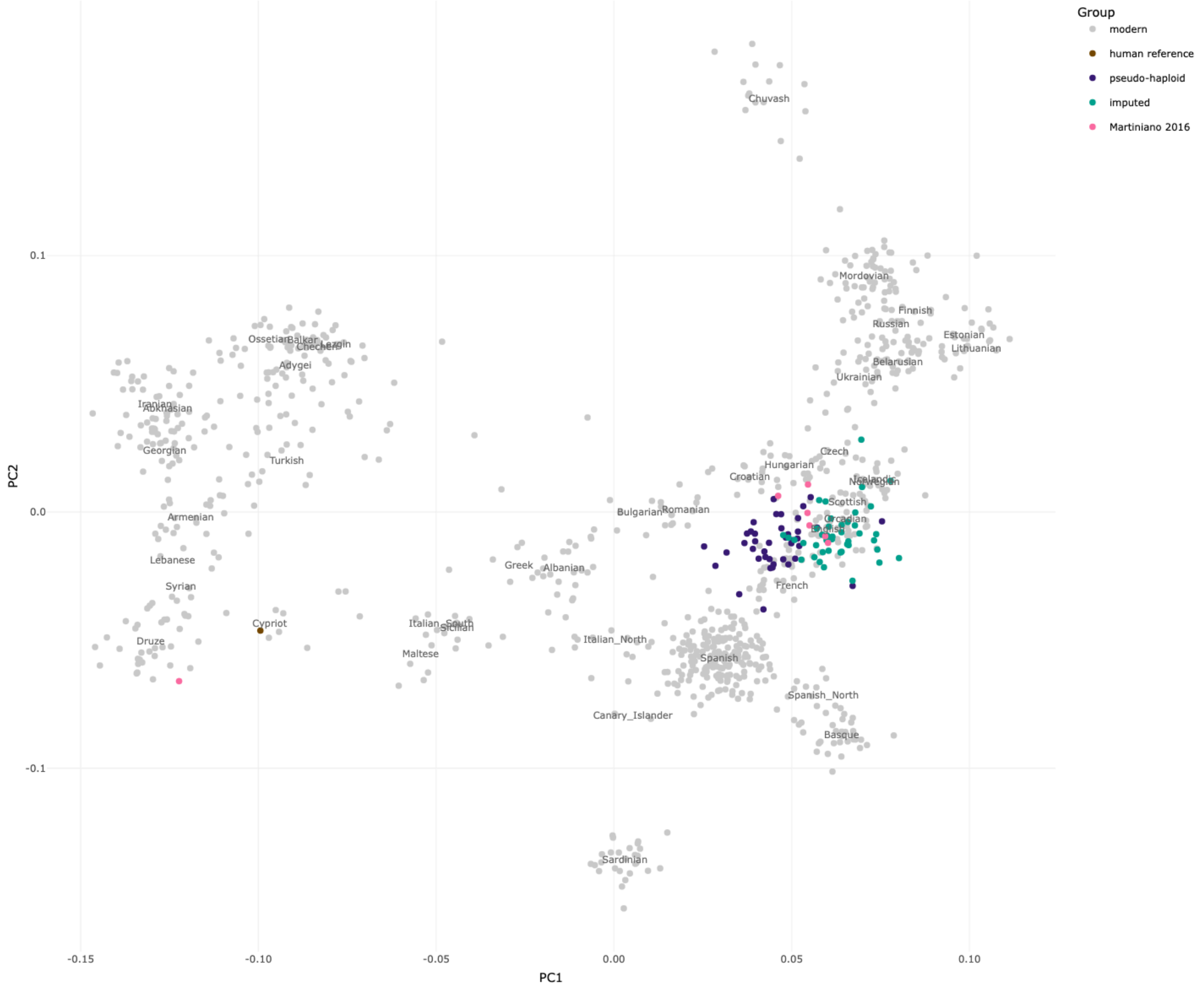

Figure S1. PNG snapshot of an Interactive PCA of haploidised and imputed genomes overlapping modern populations. Click the link here to view in html: PC plot (ggplot) html.

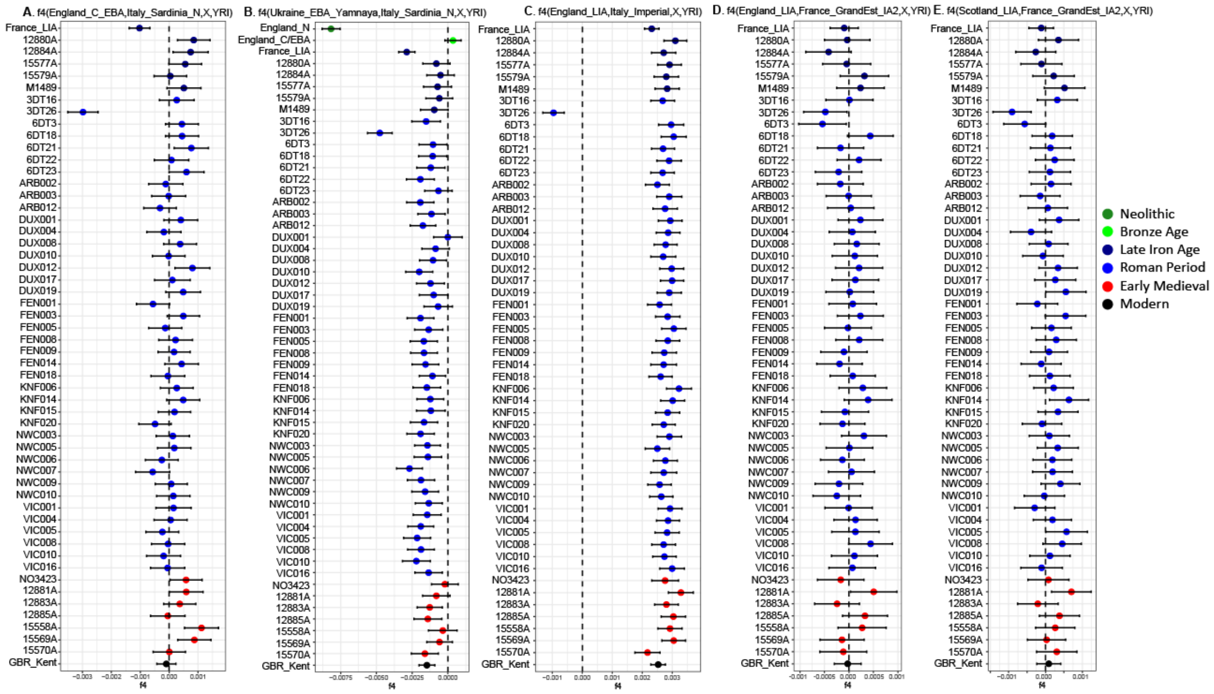

Figure S2. Genetic affinities of Roman Period individual imputed genomes to ancient populations of Europe represented by groups of ancient genomes from the Allen Ancient DNA Resource v54. GBR_Kent - 1000 Genomes Project data for individuals from Kent, England. Each plot shows the estimated *f4* value with an error range of 2 standard deviations. For contextual purposes the Roman genomes from this study (ARB002..VIC016) are shown in context of Late Iron Age (12880A..M1489), Roman (3DT16..6DT23), and Early Medieval (NO3423..15570A) genomes from previous studies (Martiniano et al. 2016 and Schiffels et al. 2016), imputed alongside with the Roman genomes of this study. 3DT26 is the long-distance migrant with likely origin in Jordan or Syria (Martiniano et al. 2016) mentioned in the text.

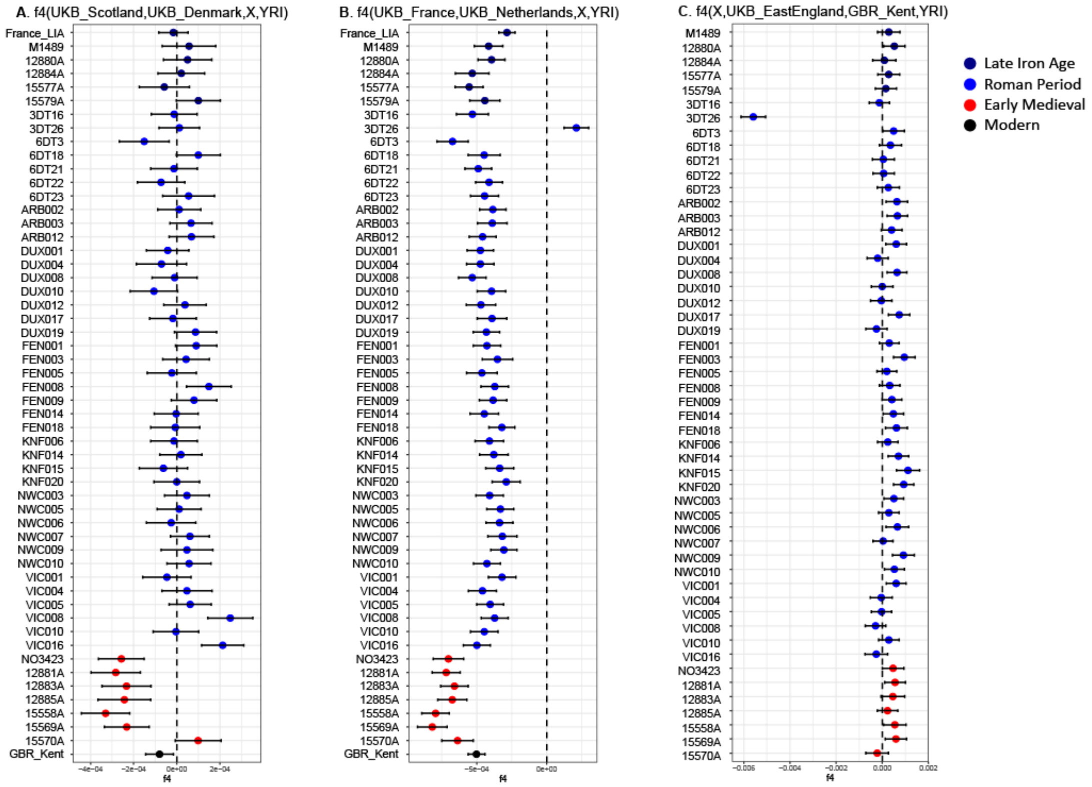

Figure S3. Genetic affinities of Roman Period individual genomes to modern populations of Europe. A-C: affinities to groups of 200 individuals from the UK Biobank born in France, Netherlands, Denmark, Scotland and East of England (UKB_EastEngland), and the subset of the 1000 Genomes Project data for individuals from Kent, England. Each plot shows the estimated *f4* value with an error range of 2 standard deviations. For contextual purposes the Roman genomes from this study (ARB002..VIC016) are shown in context of Late Iron Age (12880A..M1489), Roman (3DT16..6DT23), and Early Medieval (NO3423..15570A) genomes from previous studies (Martiniano et al. 2016 and Schiffels et al. 2016), imputed alongside with the Roman genomes of this study. 3DT26 is the long-distance migrant with likely origin in Jordan or Syria (Martiniano et al. 2016) mentioned in the text.

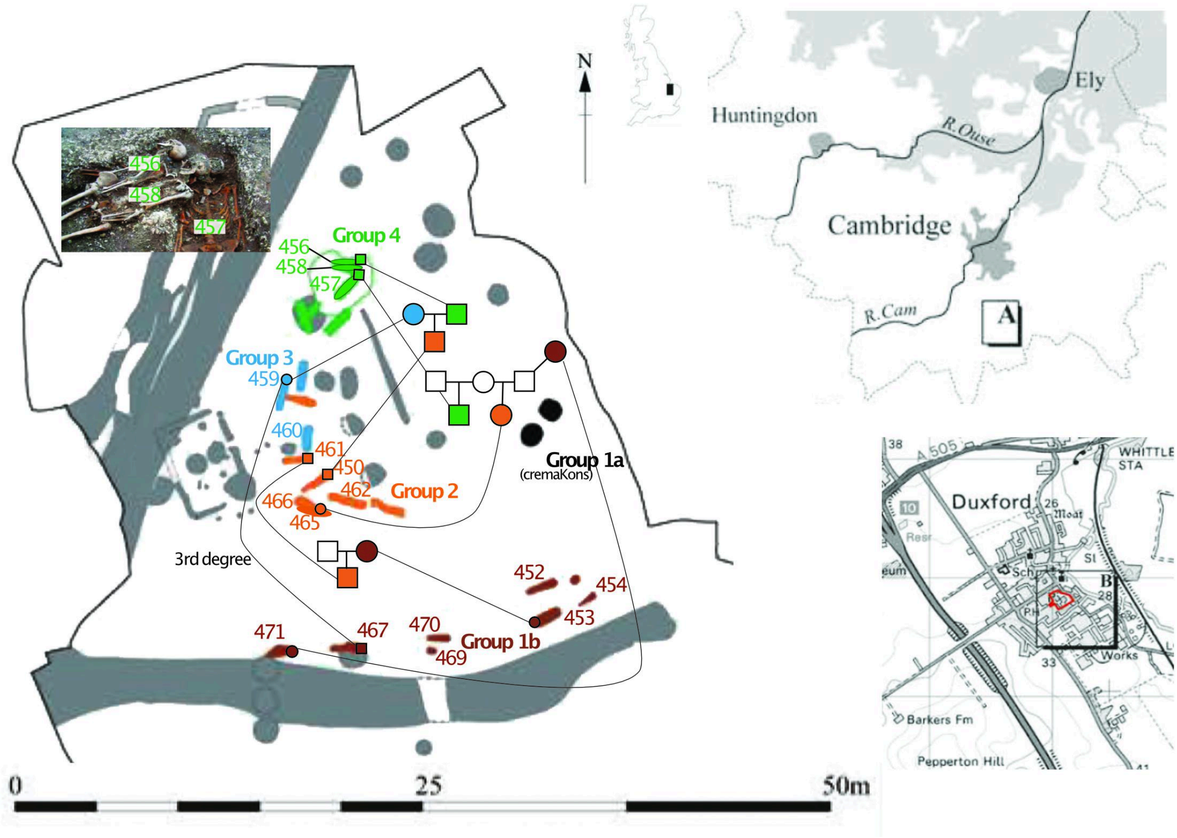

Figure S4. Map of genetic relatedness among burial groups of the Roman site of Duxford. Burial map from Alice Lyons 2011 "Life and Afterlife at Duxford, Cambridgeshire:archaeology and history in a chalkland community" East Anglian Archaeology Report No. 141, 2011 adapted with kinship findings of this study.

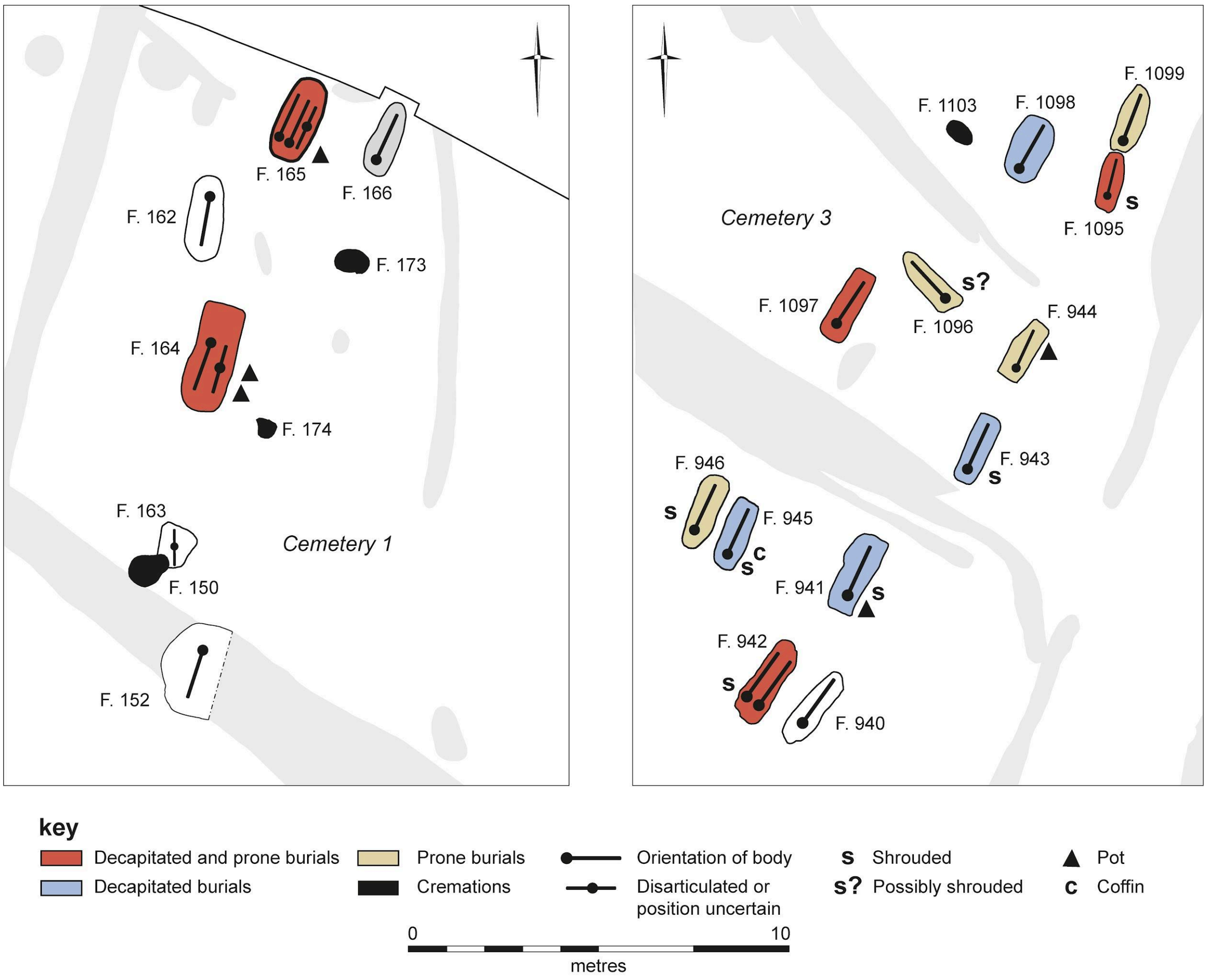

Figure S5. Knobb's Farm Cemetery 1 and 3 excavation plan. Adapted from Wiseman et al. 2021.

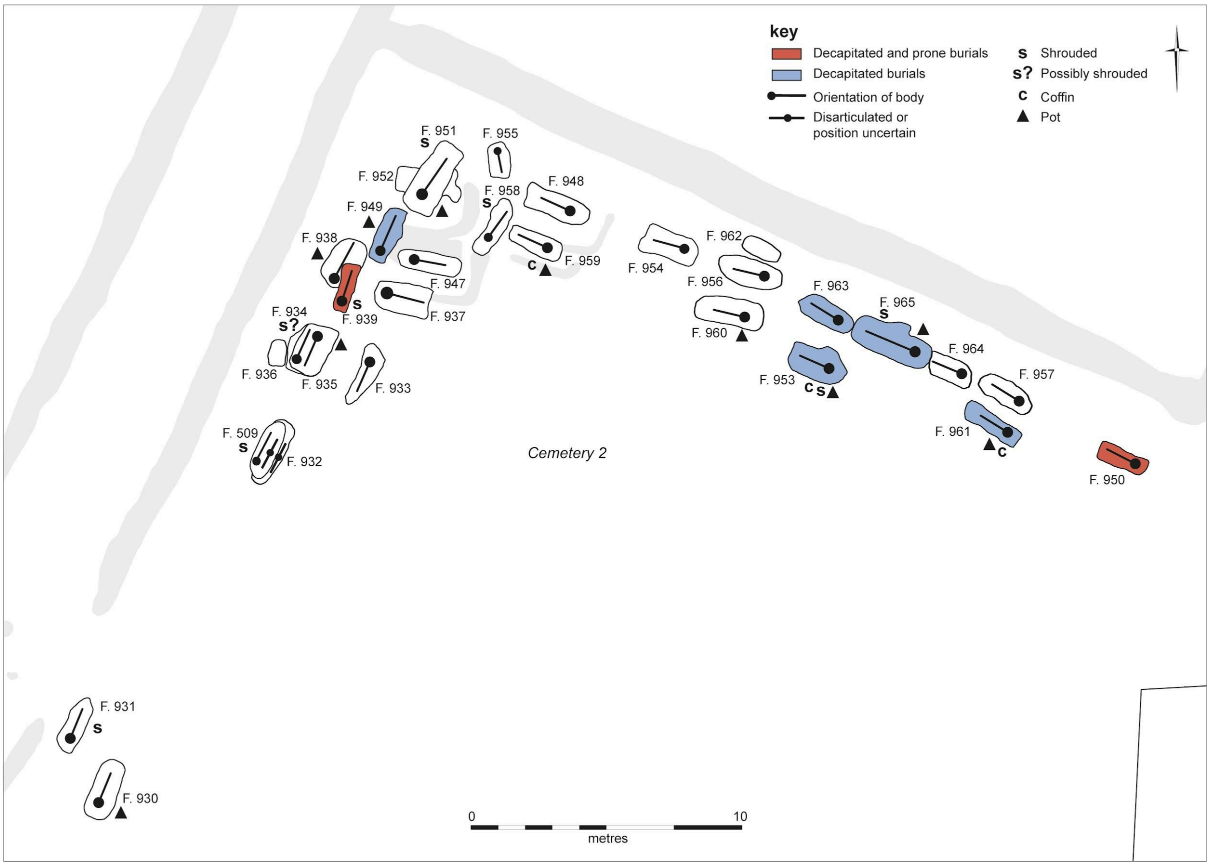

Figure S6. Knobbs' Farm Cemetery 2 excavation plan. Adapted from Wiseman et al. 2021.

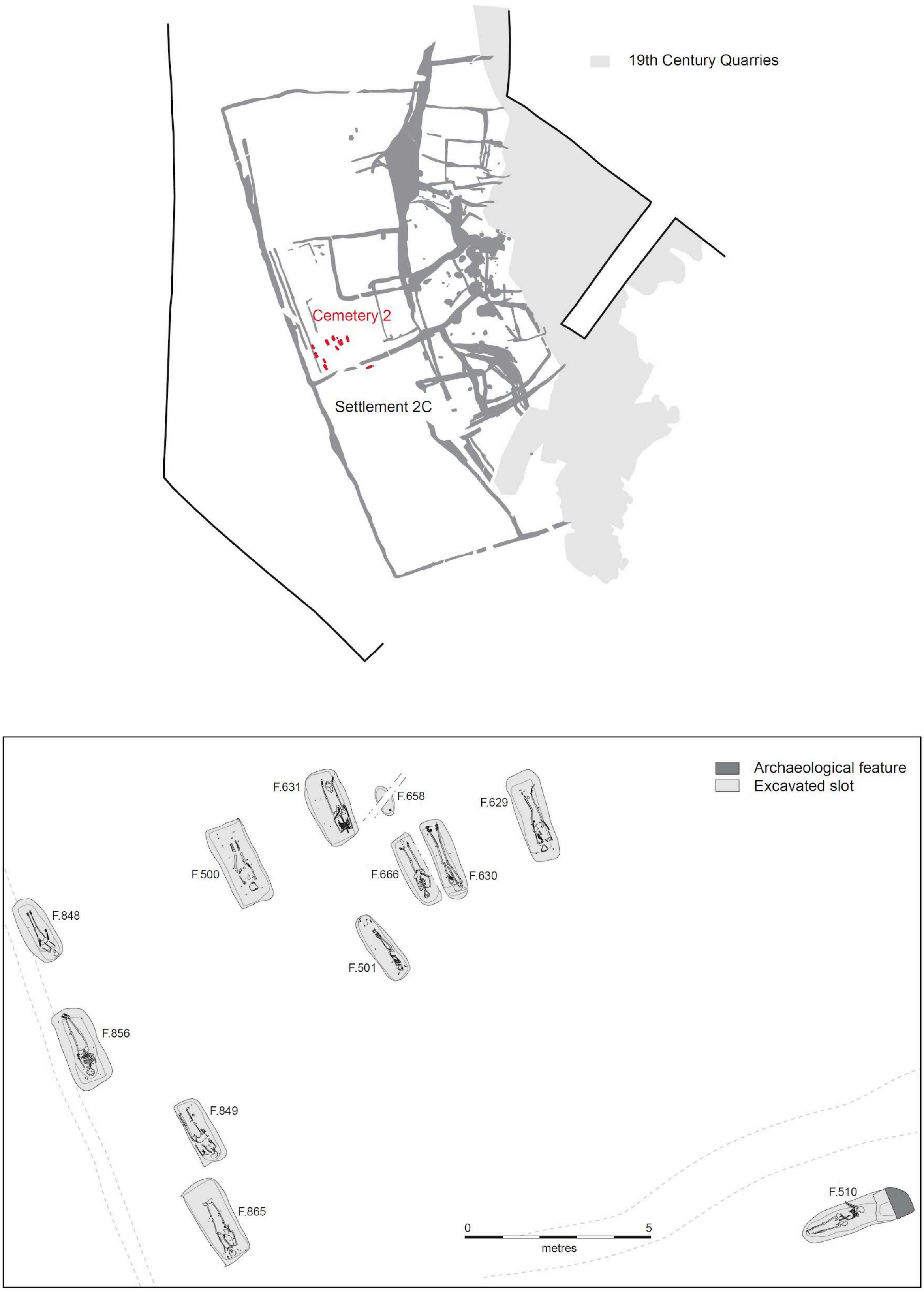

Figure S7. Northwest Cambridge Cemetery plan. From Cessford, C., & Evans, E. (2014). North West Cambridge Archaeology. University of Cambridge 2012-2013 Excavations (Report No. 3; Parts 1-3). Cambridge Archaeological Unit Report.
